# Major vascular events after first incident stroke: a population-based study

**DOI:** 10.1136/bmjno-2024-000723

**Published:** 2024-10-30

**Authors:** Rayka Malek, Salha Alasiri, Charles D A Wolfe, Abdel Douiri

**Affiliations:** 1 School of Life Course & Population Sciences, King's College London, London, UK

**Keywords:** CEREBROVASCULAR, STROKE, SUDDEN DEATH

## Abstract

**Background:**

Recent advances in stroke care have led to improvements in survival and rates of stroke recurrence. However, there is a lack of data on trends of major vascular events, and risk factors associated with non-fatal and fatal outcomes. We aim to identify demographical and clinical factors leading to incidence of subsequent major vascular events after the first-ever stroke.

**Methods:**

6051 patients’ records with first-ever stroke between 1995 and 2018 in South London, UK were analysed. Semicompeting risks models were constructed to estimate factors affecting time to incidence of recurrent stroke, myocardial infarction (MI), mortality and transitions from poststroke recurrence/MI to mortality (indirect mortality). Cumulative incidence functions were plotted for each major vascular event, stratified by stroke subtypes. All models were adjusted for age, sex, socioeconomic status, comorbidities, stroke severity and stroke subtype.

**Results:**

Five years of cumulative incidences were 9.2% (95% CI (8.4% to 10.0%)) for recurrent stroke, 4.4% (95% CI 3.9% to 5.0%) for MI, and 45% (95% CI 44% to 47%) for mortality. Prior atrial fibrillation was associated with 47% increased risk of mortality (HR=1.47 (95% CI 1.23 to 1.75)) and a previous diagnosis of MI was the strongest risk factor for poststroke MI (HR=9.17 (95% CI 6.28 to 13.39)). Stroke unit was associated with a 40% lower hazard of mortality without having a recurrent stroke/MI (HR=0.60 (95% CI 0.50 to 0.72)) and a 39% lower hazard of indirect mortality (HR=0.57 (95% CI 0.37 to 0.87)).

**Conclusion:**

Major vascular events are prevalent after stroke, particularly among those with concurrent vascular conditions. The rate of stroke recurrence plateaued in the last decade, yet MI incidence increased. Targeted strategies to control risk factors are required to reduce the incidence of a second vascular event and prevent progression to mortality in these high-risk groups.

**WHAT IS ALREADY KNOWN ON THIS TOPIC**

- Risk of major cardiovascular events (MACE) after stroke is largely preventable by preventive strategies targeting modifiable risk factors such as hypertension, diabetes mellitus, physical inactivity, diet and psychosocial factors.
- Having a non-fatal vascular event including recurrent stroke and myocardial infarction (MI) biases risk estimation of mortality substantially.

**WHAT THIS STUDY ADDS**

- Females were at lower risk of direct mortality and MI after stroke; however, there were no sex disparities in transition from a non-fatal event to mortality (indirect mortality).
- Atrial fibrillation, a risk factor for MACE, significantly increased the magnitude of risk of death following a non-fatal event.

**HOW THIS STUDY MIGHT AFFECT RESEARCH, PRACTICE OR POLICY**

- Implementation of effective preventive strategies is still suboptimal in high-risk groups.
- Providing more intensive control of risk factors as early as possible after stroke index may be required to reduce subsequent vascular incidence and prevent mortality.

## Introduction

Stroke is a major contributor to disability and a leading cause of mortality worldwide.^1^ Significant progress in both prevention and acute management over the last decades resulted in improved survival.^2 3^ Despite this improved poststroke survival, the incidence of major adverse cardiovascular events (MACE) and the burden of stroke care remains high.^4^ Mortality after recurrent stroke is high, with half of the stroke survivors dying after 5 years.^5^

The overall population attributable risk of stroke recurrence is mainly a result of modifiable risk factors such as hypertension, diabetes mellitus, physical inactivity, diet and psychosocial factors.^6^ Furthermore, the subtype of first stroke is linked to the risk of subsequent stroke and an opportunity for targeted preventive strategies.^7^ Large artery atherosclerosis and cardioembolic (CE) strokes are often linked with an extensive vascular deficits, while the small vessel occlusion (SVO) typically displays few or mild symptoms.^8^

Commonly, patients who had a stroke might be deceased either before having any recurrent stroke or myocardial infarction (MI) (direct mortality) or after (indirect mortality).^9^ Having a non-fatal event including stroke recurrence and MI biases the risk of mortality substantially.^10^,^13^ Therefore, it is important to assess the risk of MACE after the first stroke considering the transitional risk of semicompeting events.

This study aimed to investigate the association of patients’ characteristics and non-fatal events (stroke recurrence and MI) and fatal event (mortality) using semicompeting data analysis of a 23-year follow-up register of first-ever stroke. Additionally, factors leading to the transition from non-fatal events (MI/stroke) to mortality were evaluated.

## Methods

### Study population

The South London Stroke Register is an ongoing multiethnic, urban-based population register of individuals living in South London at the time of their first stroke in a defined population of inner London, including 22 electoral wards in Lambeth and Southwark.

### Case ascertainment

Standardised criteria were applied to ensure completeness of case ascertainment, including multiple overlapping sources of notification. All patients with a suspected diagnosis of stroke or transient ischaemic attack documented in different hospital-based and community-based information sources were investigated for study eligibility. Completeness of case ascertainment has been estimated at 88% by a multinomial-logit capture–recapture model using the methods described elsewhere.^14^

### Definitions

Stroke was defined according to the WHO criteria; ischaemic stroke (ICD-10 code I63), including lacunar infarction and non-lacunar infarction; intracerebral haemorrhage (I61); subarachnoid haemorrhage (I60) and unspecified stroke (I64). Recurrent stroke was defined as any hospitalised stroke events (ICD-10 I60, I61, I63, I64). MACE was defined as recurrent stroke, MI (I21) or vascular mortality (I00–99), whichever was reported in the first 1a–1c of the death certificate. Only stroke recurrences occurring 21 days after the initial event, or earlier if they were clearly in a different vascular territory, were included.^15^ MI, including STEMI and nSTEMI, was recorded from primary and secondary care patients’ electronic health records.

Pathological stroke subtypes were classified using neuroradiology or necropsy results into ischaemic stroke, primary intracerebral haemorrhage (PICH) or subarachnoid haemorrhage. Ischaemic strokes were further investigated according to two stroke subtypes: the Oxfordshire Community Stroke Project^16^ classification and the modified Trial of Org 10 172 in Acute Stroke Treatment (TOAST) classification.^17^ Assignment to each classification was done by the senior stroke consultant acting as clinical lead on the study.

### Data collection

Specially trained study nurses and field workers collected data contemporaneously from medical records and complemented with patient interview. Information on prior to stroke diagnoses of hypertension, transient ischaemic attack, atrial fibrillation (AF), MI, diabetes mellitus and other medical conditions was collected. Attendance by a specialist stroke physician during hospitalisation and whether they were seen in a stroke unit were recorded. Patients were assessed at the onset of stroke, at 3 months and annually after stroke. All follow-up assessments included in the present study were completed by 31 December 2018.

### Statistical methods

**Figure 1:**
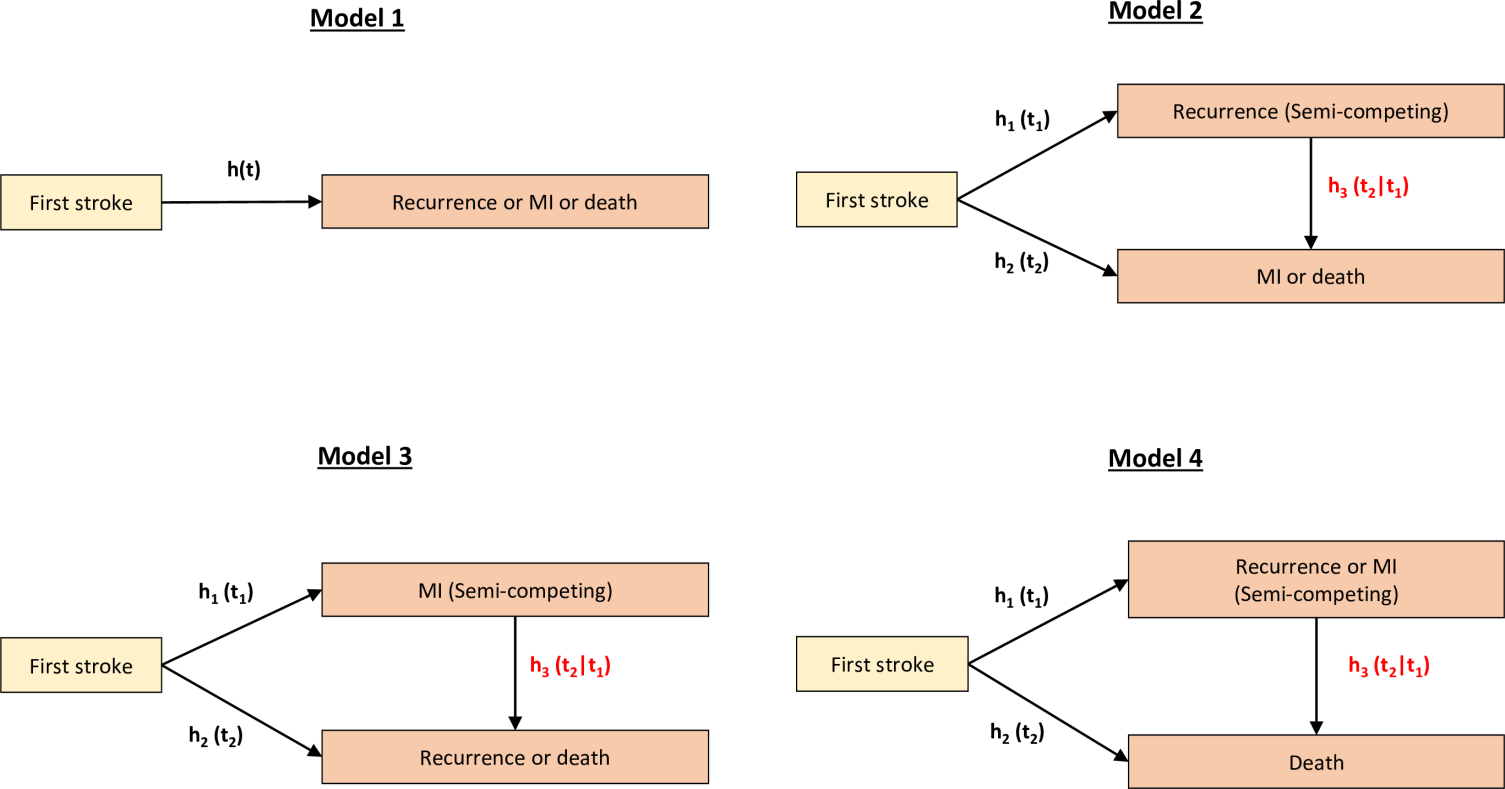
Graphical representation of statistical models proposed to analyse poststroke outcomes under the semicompeting risks framework; *h_1_*(*t_1_*), the hazard for recurrence and MI (non-fatal events) at time *t*_1_; *h_2_(t_2_*), the hazard for mortality (fatal event) at time *t*_2_; *h_3_ (t_2_|t_1_*), the hazard for death after a recurrence or MI at time *t*_2_, given that a recurrence or an MI event was observed at time *t_1_*. MI, myocardial infarction.

A description of the cohort at the time of their first stroke is reported using frequencies and percentages. Cumulative incidence of recurrent stroke, MI and mortality is reported, and details are provided in table 1 of online supplemental document. Four models were constructed to investigate associations between risk factors and outcomes (figure 1). Model 1 provided factors affecting incidence of any vascular event post first stroke using a proportional hazard Cox model. Models 2, 3 and 4 were used to study factors leading to incidence of recurrent stroke and MI (non-fatal events), factors affecting incidence of mortality (the fatal event) and finally factors associated with the transition from non-fatal events to mortality. In each model, a semicompeting risks analysis using the illness-death model was performed. Multivariable models were adjusted for age, sex, socioeconomic status, comorbidities, stroke severity and stroke subtype. Adjusted HRs with their corresponding 95% CI are presented. Analysis was conducted only on those with complete information on all covariates. To assess the robustness of the results, we performed additional analyses using multiple imputation method based on inverse probability weights. The probability of response was estimated with multivariable logistic regression including factors associated with dropping out (clinical factors at previous visits, age, socioeconomic status and race). This approach had little effect on the estimates. To assess the effect of TOAST stroke subtype, we included only index strokes since 2000 where it was collected. All statistical analyses and graphics were conducted by using RStudio V.4.2.1.

**Table 1:** Demographics of all individuals at time of initial stroke

Variable	Category	N (%)
Total		6051
Year of first stroke	1995–1999	1577 (26.1)
	2000–2004	1317 (21.8)
	2005–2009	1209 (20.0)
	2010–2014	1073 (17.7)
	2015–2018	875 (14.5)
Age	<65 years	2081 (34.4)
	65–74 years	1436 (23.7)
	75–84 years	1618 (26.7)
	85+ years	916 (15.1)
Sex	Female	2940 (48.6)
Ethnicity	White	4000 (66.1)
	Black	1528 (25.3)
	Other	378 (6.2)
	Unknown	145 (2.4)
Socioeconomic group	Non-manual	1529 (25.3)
	Manual	2669 (44.0)
	Economically inactive	1295 (21.4)
	Unknown	558 (9.2)
Stroke subtype	Ischaemic	4357 (72.0)
	Haemorrhage	1045 (17.3)
	Unknown	649 (10.7)
Oxfordshire community stroke classification	TACI	748 (12.4)
	PACI	1513 (25.0)
	POCI	700 (11.6)
	LACI	1369 (22.6)
	PICH	756 (12.5)
	SAH	289 (4.8)
	Unknown	676 (11.2)
TOAST classification of ischaemic strokes	Cardioembolic	833 (26.5)
	Large artery atherosclerosis	351 (11.2)
	SVO	809 (25.7)
	Ischaemic stroke of other causes	82 (2.6)
	Ischaemic stroke of undetermined cause	746 (23.7)
	Ischaemic stroke of multiple possible causes	126 (4.0)
	Cryptogenic (unknown cause)	198 (6.3)
	Not done (pre 2000)	1577
	Unknown	577
Risk factors (diagnosed before first stroke)		
Transient ischaemic attack	No	5111 (84.5)
	Yes	625 (10.3)
	Unknown	315 (5.2)
Hypertension	No	2010 (33.2)
	Yes	3760 (62.1)
	Unknown	281 (4.6)
Diabetes mellitus	No	4554 (75.3)
	Yes	1237 (20.4)
	Unknown	260 (4.3)
Myocardial infarction	No	5106 (84.4)
	Yes	628 (10.4)
	Unknown	317 (5.2)
Atrial fibrillation	No	4728 (78.1)
	Yes	1011 (16.7)
	Unknown	312 (5.2)
Peripheral vascular disease	No	5366 (88.7)
	Yes	254 (4.2)
	Unknown	431 (7.1)
Current smoker	No	3746 (61.9)
	Yes	1700 (28.1)
	Unknown	605 (10.0)
Stroke severity		
Glasgow coma score	Severe (<8)	793 (13.1)
	Moderate (9–12)	735 (12.1)
	Mild (13–15)	4234 (70.0)
	Unknown	289 (4.8)
Incontinence	No	3332 (55.1)
	Yes	2317 (38.3)
	Unknown	402 (6.6)
Admitted/transferred to a stroke unit	No	2002 (33.1)
	Yes	3364 (55.6)
	Not applicable	615 (10.2)
	Unknown	70 (1.2)

LACI, lacunar infarct; PACI, partial anterior circulation infarct; PICH, primary intracerebral haemorrhage; POCI, posterior circulation infarct; SAH, subarachnoid haemorrhage; SVO, small-vessel occlusion; TACI, total anterior circulation infarct; TOAST, Trial of Org 10 172 in Acute Stroke Treatment.

## Results

**Figure 2:**
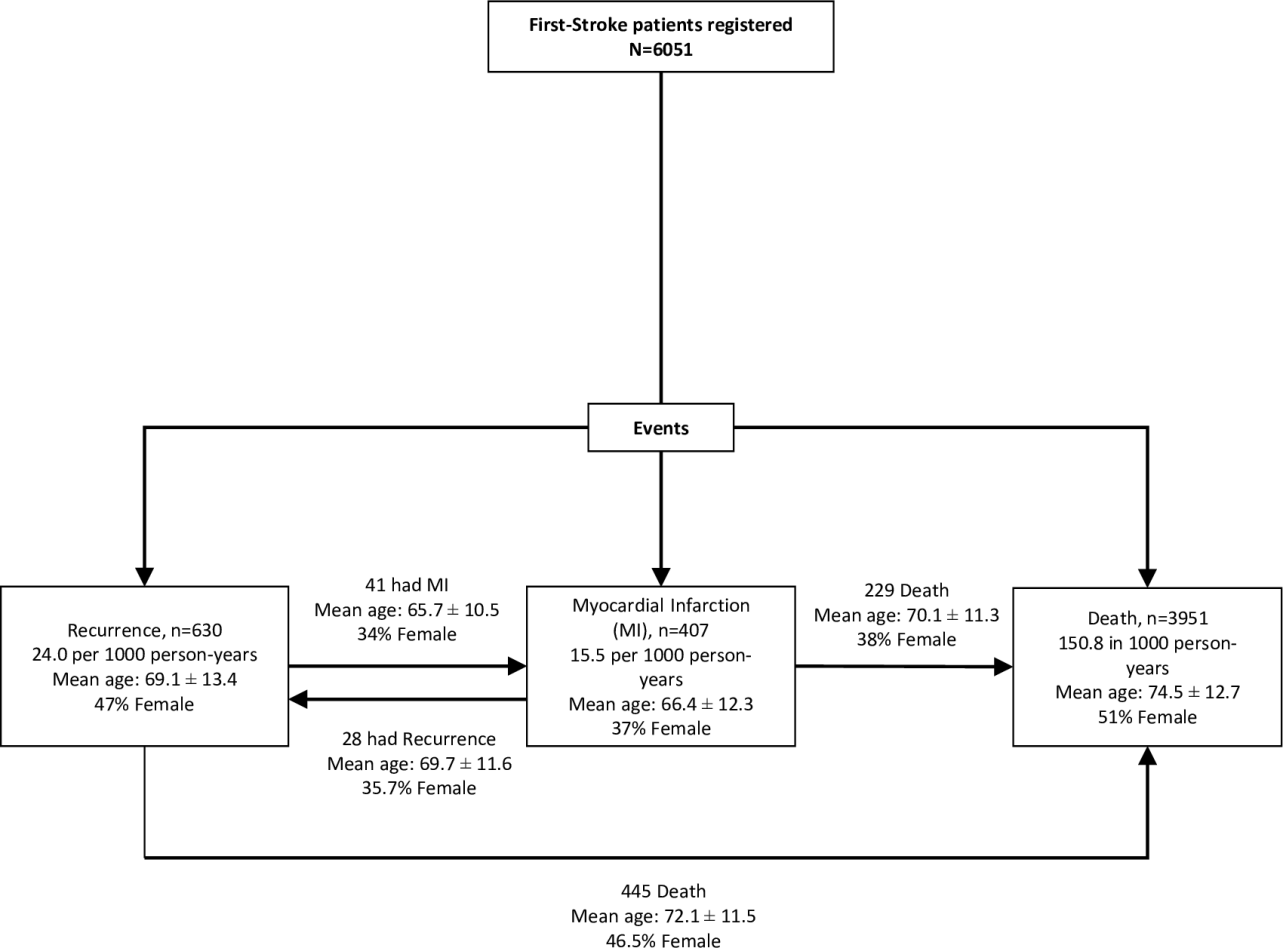
Study flow diagram illustrating frequency of events occurring post first stroke and patients’ demographics in each group.

A total of 6051 individuals were recruited for the study. The median follow-up time for observing an event (stroke recurrence, MI and mortality, whichever occurs first) was 1.9 years with a total of 26 196 person-years follow-up. Figure 2 illustrates the study flow diagram and frequency of events that occurred post first stroke.

One-third (n=2081, 34.4%) were under 65 years at the time of their first stroke, 2940 (48.6%) were female and 4000 (66.1%) were of white ethnicity. More than 70% of first strokes (n=4357) were ischaemic (12.5% PICH). Further details of the population are presented in table 1.

**Figure 3:**
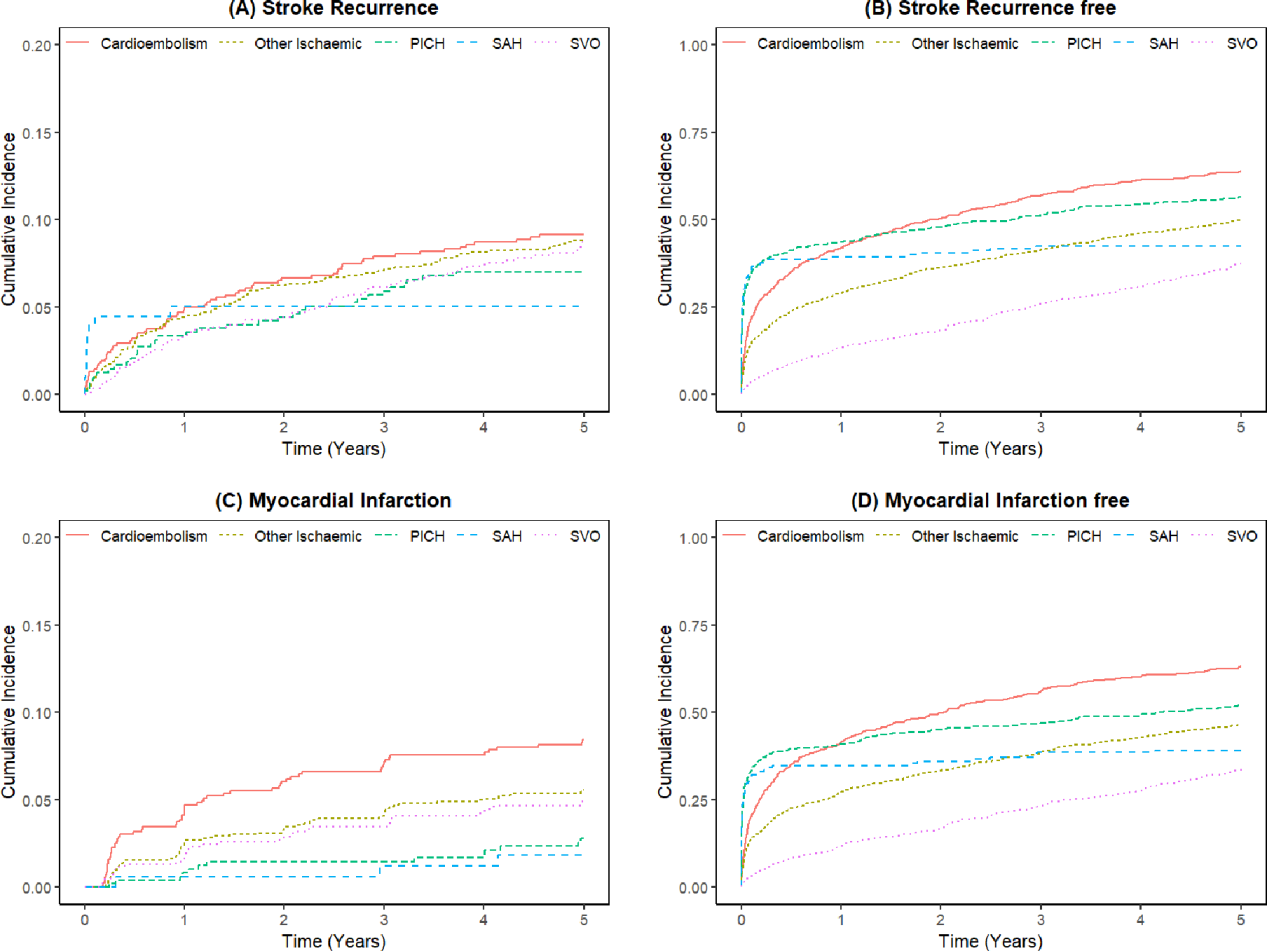
The cumulative incidence curves for each competing event by first stroke subtype. (**A**) Cumulative incidence of stroke recurrence by first stroke subtype; (**B**) cumulative incidence of recurrence or death by first stroke subtype; (**C**) cumulative incidence of myocardial infarction by first stroke subtype; (**D**) cumulative incidence of myocardial infarction or death by first stroke subtype. PICH, primary intracerebral haemorrhage; SAH, subarachnoid haemorrhage; SVO, small-vessel occlusion.

Excluding those without TOAST classification, CE stroke subtype had the highest rate of stroke recurrence, MI and mortality (figure 3). Further analysis by TOAST subtype is provided in figure 1 of online supplemental document.

### Stroke recurrence

At least one subsequent stroke occurred in 630 individuals during study period (24.0 per 1000 person-years). The overall 5-year cumulative incidence of stroke recurrence was 9.2% (95% CI 8.4%, 10.0%). Cumulative incidence of stroke recurrence at 5 years was 11% (95% CI 9.2%, 12%) for first strokes between 1995 and 1999 dropping to around 7.9% (95% CI 6.5%, 9.6%) since 2005 and then plateaued (online supplemental table 1). The adjusted results in table 2 reveal the hazard of having a subsequent stroke was lower in the later cohorts (HR for 2010–2014=0.61 (95% CI 0.40, 0.92), p=0.02).

**Table 2:** Multivariable proportional hazard models for cardiovascular free, recurrent stroke and myocardial infarction outcomes after first stroke, adjusted for variables listed, including only those with all information (N=3878)

Variable	Category	Cardiovascular free HR (95% CI)	P value	Recurrence* HR (95% CI)	P value	MI† HR (95%CI)	P value
Year of first stroke	1995–1999	1	–	1	–	1	–
	2000–2004	0.98 (0.86,1.11)	0.71	0.82 (0.59,1.14)	0.24	5.07 (2.53,10.17)	<0.001
	2005–2009	0.81 (0.71,0.94)	<0.001	0.58 (0.40,0.84)	0.004	3.56 (1.72,7.39)	0.001
	2010–2014	0.78 (0.65,0.92)	<0.001	0.61 (0.40,0.92)	0.02	4.39 (2.04,9.44)	<0.001
	2015–2018	0.71 (0.58,0.88)	<0.001	0.42 (0.25,0.70)	0.001	5.41 (2.36,12.39)	<0.001
Age	<65 years	1	–	1	–	1	–
	65–74 years	1.84 (1.60,2.12)	<0.001	1.38 (1.01,1.89)	0.04	1.33 (0.86,2.06)	0.19
	75–84 years	2.52 (2.20,2.90)	<0.001	1.76 (1.28,2.42)	<0.001	1.41 (0.89,2.22)	0.14
	85+ years	3.89 (3.32,4.55)	<0.001	2.31 (1.53,3.49)	<0.001	1.92 (1.02,3.60)	0.04
Sex	Male	1	–	1	–	1	–
	Female	0.97 (0.88,1.06)	0.51	1.13 (0.89,1.43)	0.31	0.77 (0.54,1.09)	0.15
Ethnicity	White	1	–	1	–	1	–
	Black	0.85 (0.75,0.96)	0.01	1.21 (0.92,1.60)	0.18	0.57 (0.37,0.87)	0.01
	Other	0.74 (0.60,0.91)	0.01	1.30 (0.83,2.04)	0.25	0.61 (0.31,1.21)	0.16
Socioeconomic group	Non-manual	1	–	1	–	1	–
	Manual	1.14 (1.03,1.27)	0.01	1.14 (0.88,1.48)	0.32	1.16 (0.79,1.70)	0.45
	Economically inactive	1.68 (1.46,1.93)	<0.001	1.34 (0.89,2.01)	0.16	1.11 (0.64,1.92)	0.72
Stroke subtype	Infarct	1	–	1	–	1	–
	PICH‡	0.97 (0.85,1.12)	0.69	1.55 (1.07,2.23)	0.02	0.76 (0.40,1.45)	0.41
	SAH§	0.76 (0.56,1.03)	0.08	0.50 (0.19,1.29)	0.15	0.22 (0.03,1.68)	0.15
Transient ischaemic attack	No	1	–	1	–	1	–
	Yes	1.02 (0.90,1.16)	0.77	1.33 (0.97,1.83)	0.07	1.02 (0.62,1.68)	0.94
Hypertension	No	1	–	1	–	1	–
	Yes	1.03 (0.93,1.13)	0.61	1.18 (0.91,1.53)	0.21	1.64 (1.08,2.49)	0.02
Diabetes mellitus	No	1	–	1	–	1	–
	Yes	1.19 (1.06,1.33)	<0.001	1.19 (0.90,1.57)	0.23	1.81 (1.24,2.64)	0.002
Myocardial infarction	No	1	–	1	–	1	–
	Yes	1.41 (1.24,1.60)	<0.001	1.46 (1.02,2.10)	0.04	9.17 (6.28,13.39)	<0.001
Atrial fibrillation	No	1	–	1	–	1	–
	Yes	1.39 (1.25,1.55)	<0.001	1.98 (1.47,2.67)	<0.001	0.97 (0.61,1.56)	0.92
Peripheral vascular disease	No	1	–	1	–	1	–
	Yes	1.28 (1.04,1.57)	0.02	1.32 (0.76,2.28)	0.33	1.44 (0.75,2.77)	0.27
Smoker	No	1	–	1	–	1	–
	Yes	1.16 (1.04,1.29)	0.01	1.32 (1.01,1.72)	0.04	1.68 (1.15,2.45)	0.007
Glasgow Coma Score	Mild	1		1		1	
	Moderate/severe	1.83 (1.63,2.04)	<0.001	1.30 (0.85,2.01)	0.23	2.36 (1.35,4.13)	0.003
Incontinence	No	1	–	1	–	1	–
	Yes	1.71 (1.55,1.90)	<0.001	1.54 (1.14,2.07)	0.005	1.65 (1.06,2.56)	0.03
Admitted/transferred to stroke unit	No	1	–	1	–	1	–
	Yes	0.74 (0.66,0.83)	<0.001	0.80 (0.59,1.09)	0.16	1.04 (0.64,1.71)	0.86
	Not applicable	0.67 (0.56,0.81)	<0.001	1.23 (0.85,1.78)	0.27	0.77 (0.37,1.61)	0.49

* Obtained from *h*_*1*_*(t*_*1*_*)* in model 2

† obtained from *h*_*2*_*(t*_*2*_*)* in model 3

‡ PICH=Primary Intracerebral Haemorrhage

§ SAH=Subarachnoid Haemorrhage

PICH, primary intracerebral haemorrhage; SAH, subarachnoid haemorrhage.

Older age (HR for 85+ years=2.31 (95% CI 1.53, 4.49), p<0.001), having PICH subtype (HR=1.55 (95% CI 1.07, 2.23), p=0.02), a history of MI (HR=1.46 (95% CI 1.02, 2.10), p=0.04), a history of AF (HR=1.98 (95% CI 1.47, 2.67), p<0.001), smoking (HR=1.32 (95% CI 1.01, 1.72), p=0.04) and a more severe stroke that resulted in incontinence (HR=1.54 (95% CI 1.14, 2.07), p=0.005) were risk factors for a recurrent stroke after full adjustment (table 2).

### MI after stroke

Poststroke, 407 patients have had a diagnosis of MI within the study period (15.5 per 1000 person-years). The 5-year cumulative incidence of MI was 4.40% (3.90%, 5.00%). Cumulative incidence of MI at 5 years was 1.0% (0.61%, 1.6%) for first strokes between 1995 and 1999 increasing to around 5.8% (4.4%, 7.4%) between 2010 and 2014 and continued to increase thereafter (online supplemental table 1). After adjusting for all other covariates (table 2), later cohorts were associated with increased hazard of MI (eg, hazard of having an MI in those who had their first stroke in 2010–2014 was more than fourfold higher than those in 1995–1999 cohort, p<0.001).

A previous MI was the strongest clinical risk factor for having an MI after stroke (HR=9.17 (95% CI 6.28, 13.39), p<0.001). Other risk factors included older age (HR for 85+ years=1.92 (95% CI 1.02, 3.60), p=0.04), prior hypertension (HR=1.64 (95% CI 1.08, 2.49), p=0.02), prior diabetes (HR=1.81 (95% CI 1.24, 2.64), p=0.002), having moderated/severe Glasgow Coma Score (HR=2.36 (95% CI 1.35, 4.13), p=0.003), and a more severe stroke that resulted in incontinence (HR=1.65 (95% CI 1.06, 2.56), p=0.03). Individuals of black ethnicity were at lower risk of an MI than those of white ethnicity (HR=0.57 (95% CI 0.37, 0.87), p=0.01).

### Cardiovascular (MI/stroke) free survival after stroke

Older age (HR for 85+ years=3.89 (95% CI 3.32, 4.55), p<0.001), manual (HR=1.14 (95% CI 1.03, 1.27), p=0.01) and inactive socioeconomic status compared with non-manual (HR=1.68 (95% CI 1.46, 1.93), p<0.001) increased the risk of having an MACE. Additionally, a previous diagnosis of diabetes (HR=1.19 (95% CI 1.06, 1.33), p<0.001), a prior MI (HR=1.41 (95% CI 1.24, 1.60), p<0.001), a prior AF (HR=1.39 (95% CI 1.25, 1.55), p<0.001) and a prior peripheral vascular disease (HR=1.28 (95% CI 1.04, 1.57), p=0.02) were clinical risk factors of MACE (table 2). Other risk factors included smoking (HR=1.16 (95% CI 1.04, 1.29), p=0.01), having moderated/severe Glasgow Coma Score (HR=1.83 (95% CI 1.63, 2.04), p<0.001) and incontinence (HR=1.71 (95% CI 1.55, 1.90), p<0.001) (table 2). Being admitted/transferred to a stroke unit at the time of first stroke illustrated approximately 30% reduction in risks of MACE (p<0.001).

### Direct mortality

3297 patients died during the study period without experiencing a recurrent stroke or MI (125.9 per 1000 person-years). The cumulative incidence of 5-year direct mortality was 45.00% (44.00%, 47.00%). After adjusting for all covariates, the hazard of direct mortality after having first stroke decreased in all cohorts after 2000s, reaching HR of 0.25 (95% CI 0.18, 0.35) in 2015–2018 (table 3, p<0.001).

**Table 3:** Multivariable proportional hazard models for mortality after first stroke, adjusted for variables listed, including only those with all information (N=3878)

Variable	Category	Mortality before MI/recurrence* HR (95% CI)	P value	Mortality after MI/recurrence† HR (95% CI)	P value
Year of first stroke	1995–1999	1	–	1	–
	2000–2004	0.94 (0.77, 1.15)	0.55	1.11 (0.67, 1.84)	0.69
	2005–2009	0.71 (0.57, 0.89)	0.002	1.27 (0.72, 2.27)	0.41
	2010–2014	0.58 (0.44, 0.75)	<0.001	0.87 (0.44, 1.68)	0.68
	2015–2018	0.25 (0.18, 0.35)	<0.001	0.45 (0.20, 0.97)	0.04
Age	<65 years	1	–	1	–
	65–74 years	3.12 (2.48, 3.91)	<0.001	3.23 (1.94, 5.38)	<0.001
	75–84 years	4.93 (3.92, 6.20)	<0.001	6.54 (3.92, 10.91)	<0.001
	85+ years	8.85 (6.80, 11.51)	<0.001	12.75 (6.88, 23.64)	<0.001
Sex	Male	1	–	1	–
	Female	0.86 (0.75, 0.99)	<0.001	1.06 (0.76, 1.49)	0.73
Ethnicity	White	1	–	1	–
	Black	0.68 (0.56, 0.82)	<0.001	0.98 (0.64, 1.51)	0.93
	Other	0.55 (0.39, 0.77)	0.001	1.19 (0.60, 2.34)	0.62
Socioeconomic group	Non-manual	1	–	1	–
	Manual	1.19 (1.00, 1.40)	0.05	1.63 (1.11, 2.38)	0.01
	Economically inactive	2.54 (2.02, 3.18)	<0.001	1.77 (0.99, 3.15)	0.05
Stroke subtype	Infarct	1	–	1	–
	PICH‡	1.23 (0.97, 1.55)	0.08	0.65 (0.36, 1.16)	0.15
	SAH§	1.18 (0.77, 1.83)	0.45	0.38 (0.04, 3.28)	0.39
Transient ischaemic attack	No	1	–	1	–
	Yes	0.98 (0.79, 1.21)	0.84	0.87 (0.56, 1.36)	0.54
Hypertension	No	1	–	1	–
	Yes	0.99 (0.85, 1.16)	0.93	1.03 (0.70, 1.51)	0.88
Diabetes mellitus	No	1	–	1	–
	Yes	1.19 (0.99, 1.43)	0.06	1.19 (0.81, 1.74)	0.37
Myocardial infarction	No	1	–	1	–
	Yes	1.35 (1.08, 1.69)	0.01	0.90 (0.60, 1.34)	0.61
Atrial fibrillation	No	1	–	1	–
	Yes	1.47 (1.23, 1.75)	<0.001	1.65 (1.12, 2.44)	0.01
Peripheral vascular disease	No	1	–	1	–
	Yes	1.36 (0.97, 1.91)	0.08	1.34 (0.67, 2.71)	0.41
Smoker	No	1	–	1	–
	Yes	1.12 (0.94, 1.33)	0.20	1.18 (0.80, 1.74)	0.40
Glasgow Coma Score	Mild	1	–	1	
	Moderate/severe	3.75 (3.00, 4.70)	<0.001	1.81 (1.06, 3.07)	0.03
Incontinence	No	1	–	1	–
	Yes	3.00 (2.48, 3.63)	<0.001	2.22 (1.50, 3.28)	<0.001
Admitted/transferred to stroke unit	No	1	–	1	–
	Yes	0.60 (0.50, 0.72)	<0.001	0.61 (0.38, 0.97)	0.04
	Not applicable	0.41 (0.23, 0.55)	<0.001	0.80 (0.47, 1.36)	0.41

* Obtained from *h*_*2*_*(t*_*2*_*)* in model 4

† Obtained from *h*_*3*_*(t*_*2*_*|t*_*1*_*)* in model 4

‡ PICH=Primary Intracerebral Haemorrhage

§ SAH=Subarachnoid Haemorrhage

PICH, primary intracerebral haemorrhage; SAH, subarachnoid haemorrhage.

Risk factors associated with higher hazard of direct mortality were older age (HR for 85+ years group=8.85 (95% CI 6.80, 11.51), p<0.001), being economically inactive (HR=2.54 (95% CI 2.02, 3.18), p<0.001) at the time of the index stroke, a prior MI (HR=1.35 (95% CI 1.08, 1.69), p=0.01), a previous diagnosis of AF (HR=1.47 (95% CI 1.23, 1.75), p<0.001), a moderate/severe Glasgow Coma Score (HR=3.75 (95% CI 3.00, 4.70), p<0.001) and having incontinence (HR=3.00 (95% CI 2.48, 3.63), p<0.001). Females and individuals of black ethnicity had 14% and 32% reduced risk of direct mortality, respectively (p<0.001) (table 3). Moreover, admission/transfer to a stroke unit was associated with better survival (HR=0.60 (95% CI 0.50, 0.72), p<0.001).

### Indirect mortality

Among patients who experienced a non-fatal cardiovascular event, 622 patients died (86.9 per 1000 person-years). The 2015–2018 cohort was at a 55% lower risk of mortality compared with the 1995–1999 cohort (HR=0.45 (95% CI 0.20, 0.97), p=0.04). Admission/transfer to a stroke unit at the time of index stroke was associated with a 40% lower risk of mortality after a non-fatal event (table 3, p=0.04).

Risk factors for indirect mortality included being older (HR for 85+ years group=12.75 (95% CI 6.88, 23.64), p<0.001), manual socioeconomic status (HR=1.63 (95% CI 1.11, 2.38), p=0.01), a previous diagnosis of AF (HR=1.65 (95% CI 1.12, 2.44), p=0.01), a moderate/severe Glasgow Coma Score (HR=1.81 (95% CI 1.06, 3.07), p=0.03) and incontinence (HR=2.22 (95% CI 1.50, 3.28), p<0.001)

## Discussion

In this large population-based study, factors affecting the incidence of subsequent stroke, MI and mortality were investigated using a semicompeting risks framework, providing unbiased incidence estimations as well as providing insight into risk factors and transitions from non-fatal vascular events to mortality.

The observed 5-year rate of recurrent stroke was 10.4% (630/6051) in our data. When accounting for death using competing risks to calculate cumulative incidence, the 5-year rate was estimated at 9.2% (8.4%–10.0%) which is lower than the Rotterdam study.^3^ This may be due to the older age of their study population (around 30% above 85 years). The Perth community stroke study also reported a higher cumulative incidence but in a different time period two decades ago,^18^ which could be explained by different secondary preventive strategies and risk exposure.

One of the most significant findings emerging from this study is that stroke recurrence and mortality rates decreased in recent years, while MI rates increased. The reduction in recurrent stroke rates could possibly indicate that advances in secondary prevention strategies have only recently succeeded in lowering the risk of recurrent stroke. Subsequent to the increased survival after the initial stroke, patients are at higher risk of MI. Another explanation for the rising trend of MI could be improvements in the diagnosis of MI. Similarly, a recent large population-based study in Scotland using a multistate model reported a drop in risk of stroke recurrence after the first stroke but the incidence of MI had risen in recent years.^13^

Consistent with age-related survival disparities reported in other studies,^19 20^ we found that age >65 years was associated with a higher risk of stroke recurrence and mortality. Black ethnicity had a protective association with MI and mortality after first stroke. This could be explained by findings from our previous SLSR study, which showed that patients from black ethnic groups were more likely to be admitted to a hospital or to a stroke unit and get access to rehabilitation and follow-up services.^21^ However, other studies showed the opposite: black minorities are less likely to receive recommended acute interventions compared with their white counterparts, resulting in increased mortality.^22^

Previous studies have reported inconsistent results regarding sex differences in stroke prognosis.^23^ A recent systematic review^24^ reported that compared with men, women tend to have better survival but poorer outcomes including stroke recurrence. Our data consistently supported that females were at 24% lower risk of mortality without having a recurrent stroke or MI; however, no differences were observed for indirect mortality. Similar to a recent study,^3^ our data did not support these sex disparities in terms of recurrent stroke.

Comorbidities including a previous diagnosis of hypertension, diabetes and MI were associated with a higher risk of poststroke MACE incidence similar to a Swedish cohort^25^ and a recent meta-analysis.^26^ Moreover, those who had a diagnosis of AF prior to their first stroke have been found to be at a higher risk of stroke recurrence and mortality. This could be justified by poor adherence or access to anticoagulation treatments. Previously, SLSR data showed 60% of AF patients were not treated with anticoagulants^21^ and more recent published reports estimated that this figure is down to 46% in the UK^27^ supporting our hypothesis.

Furthermore, admission to stroke unit at the time of index stroke contributed to decreased risk of both direct and indirect mortality. Being admitted/transferred to a stroke unit could decrease the risk of direct mortality by 40%. Also, our analysis indicated a reduced risk of progression from the recurrent stroke/MI stage to the mortality stage. A German cohort reported that treatment in a stroke unit was associated with lower mortality, hence care at a specialised centre should be provided for acute strokes whenever possible.^28^

This study had several strengths, including the prospective population-based cohort study design, the large number of stroke survivors, detailed data on stroke types, complete long-term follow-up, detailed outcomes and robust statistical methodology. The community-based setting of our study avoids significant selection bias that results from hospital-based studies and is, therefore, more accurate for determining MACE risk in the general population. However, the study also had some limitations; there may be other confounding factors that are not included in this analysis. We have considered only baseline risk factors of cardiovascular events, and we have no information on patients’ adherence to drugs, which may attenuate the effect of treatments. Further analyses investigating the impact of secondary prevention measures would be of interest.

In conclusion, the rate of stroke recurrence decreased until 2005 and plateaued ever since, yet MI incidence increased. Admission to a stroke unit significantly prolonged survival, confirming that care provision at specialised centres would potentially benefit patients’ survival. Providing more intensive control of risk factors is still suboptimal and may be required to reduce the occurrence of recurrent stroke/MI and prevent progression to mortality even among high-risk groups with other vascular conditions.

## Supplementary material

10.1136/bmjno-2024-000723 online supplemental file 1

## Data Availability

Data are available on reasonable request.
