# Supplementary material for: Major vascular events after first incident stroke: a population-based study
Source: BMJ Neurol Open. 2024 Oct 30;6(2):e000723. doi: 10.1136/bmjno-2024-000723 (PMC11529573; doi:10.1136/bmjno-2024-000723)
Supplement: online supplemental file 1 [file bmjno-6-2-s001.pdf]

Incidence of events post stroke

The cumulative incidence of each event occurring by 1 and 5 years were estimated, taking other competing events into account:

Table 1: Unadjusted cumulative Incidence of stroke recurrence, myocardial infarction, and mortality after stroke, by year of first stroke, % (95% Confidence Interval)

Year of 1st stroke	1-year	5-year
**Stroke recurrence**		
1995-1999	5.5% (4.5%, 6.8%)	11% (9.2%, 12%)
2000-2004	4.7% (3.6%, 5.9%)	8.2% (6.8%, 9.8%)
2005-2009	3.5% (2.6%, 4.7%)	7.9% (6.5%, 9.6%)
2010-2014	3.3% (2.3%, 4.5%)	8.5% (6.9%, 10%)
2015-2018	6.4% (4.4%, 8.8%)	-
**Myocardial infarction**		
1995-1999	0.45% (0.20%, 0.89%)	1.0% (0.61%, 1.6%)
2000-2004	1.3% (0.79%, 2.0%)	4.6% (3.5%, 5.8%)
2005-2009	1.3% (0.80%, 2.1%)	4.6% (3.5%, 6.0%)
2010-2014	2.1% (1.3%, 3.1%)	5.8% (4.4%, 7.4%)
2015-2018	9.7% (7.3%, 13%)	-
**Mortality**		
1995-1999	37% (35%, 40%)	55% (52%, 57%)
2000-2004	34% (32%, 37%)	47% (44%, 50%)
2005-2009	25% (23%, 28%)	42% (39%, 45%)
2010-2014	20% (17%, 22%)	37% (34%, 40%)
2015-2018	26% (23%, 30%)	-

TOAST stroke subtype

The adjusted model (Figure 1) illustrated those who had cardioembolic stroke were at 88% (P=0.01), 4-fold (P<0.001), and 60% (P=0.001) higher hazard of having a subsequent stroke, an MI, and mortality compared to small-vessel occlusion (SVO) respectively. Moreover, PICH was associated with 92% (P=0.02) and 72% (P=0.002) higher hazard of having a subsequent stroke and mortality respectively. After having a non-fatal event (recurrence and/or MI), the risk of mortality was significantly lower in individuals with PICH first-stroke (HR (95% CI)=0.35 (0.16,0.76), P=0.01).

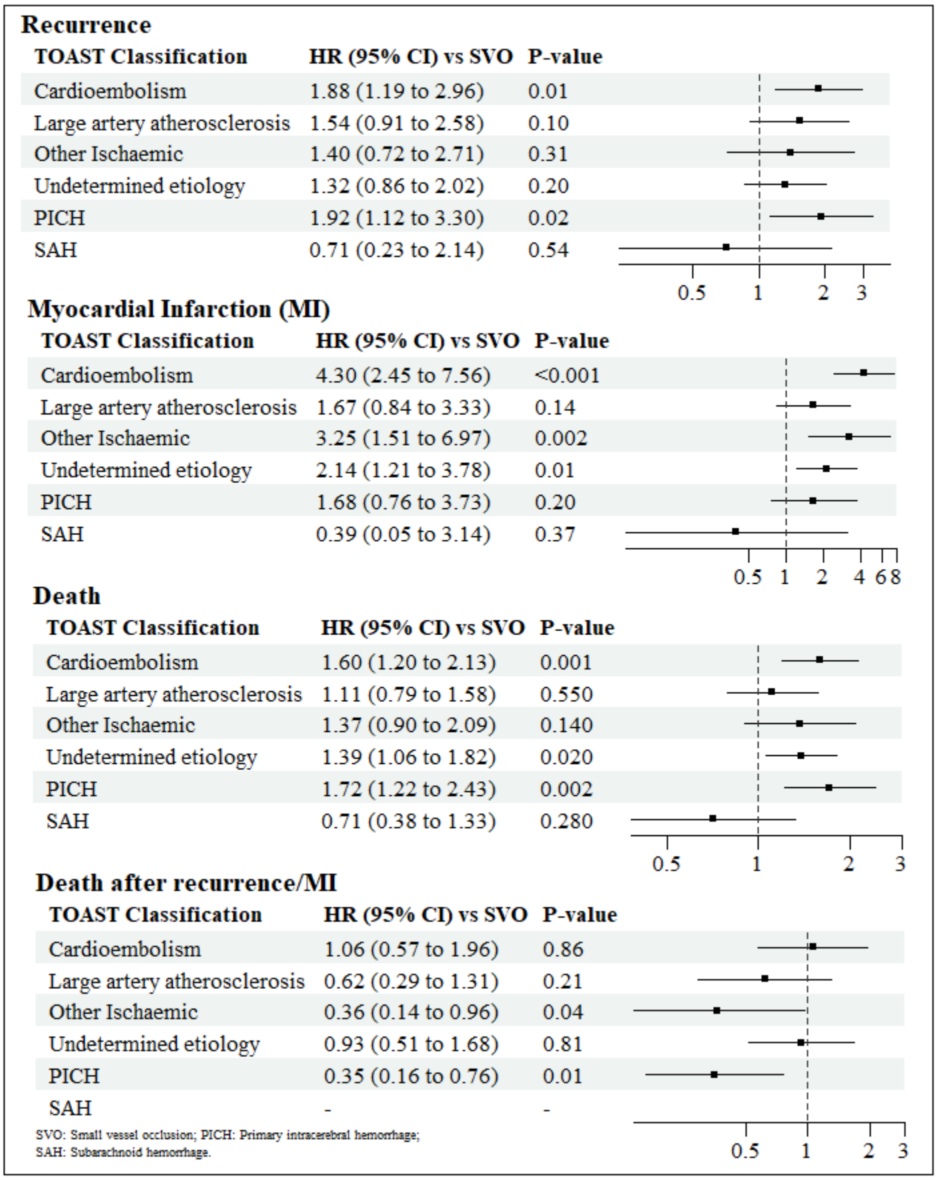

Recurrence

TOAST Classification	HR (95% CI) vs SVO	P-value
Cardioembolism	1.88 (1.19 to 2.96)	0.01
Large artery atherosclerosis	1.54 (0.91 to 2.58)	0.10
Other Ischaemic	1.40 (0.72 to 2.71)	0.31
Undetermined etiology	1.32 (0.86 to 2.02)	0.20
PICH	1.92 (1.12 to 3.30)	0.02
SAH	0.71 (0.23 to 2.14)	0.54

Myocardial Infarction (MI)

TOAST Classification	HR (95% CI) vs SVO	P-value
Cardioembolism	4.30 (2.45 to 7.56)	<0.001
Large artery atherosclerosis	1.67 (0.84 to 3.33)	0.14
Other Ischaemic	3.25 (1.51 to 6.97)	0.002
Undetermined etiology	2.14 (1.21 to 3.78)	0.01
PICH	1.68 (0.76 to 3.73)	0.20
SAH	0.39 (0.05 to 3.14)	0.37

Death

TOAST Classification	HR (95% CI) vs SVO	P-value
Cardioembolism	1.60 (1.20 to 2.13)	0.001
Large artery atherosclerosis	1.11 (0.79 to 1.58)	0.550
Other Ischaemic	1.37 (0.90 to 2.09)	0.140
Undetermined etiology	1.39 (1.06 to 1.82)	0.020
PICH	1.72 (1.22 to 2.43)	0.002
SAH	0.71 (0.38 to 1.33)	0.280

Death after recurrence/MI

TOAST Classification	HR (95% CI) vs SVO	P-value
Cardioembolism	1.06 (0.57 to 1.96)	0.86
Large artery atherosclerosis	0.62 (0.29 to 1.31)	0.21
Other Ischaemic	0.36 (0.14 to 0.96)	0.04
Undetermined etiology	0.93 (0.51 to 1.68)	0.81
PICH	0.35 (0.16 to 0.76)	0.01
SAH	-	-

SVO: Small vessel occlusion; PICH: Primary intracerebral hemorrhage;
SAH: Subarachnoid hemorrhage.

Figure 1: Adjusted hazard ratio of TOAST stroke subtype for all events after first stroke
